# Reproductive coercion experienced by women living with HIV – a global scoping review

**DOI:** 10.1080/26410397.2025.2588004

**Published:** 2026-02-03

**Authors:** Althea Wolfe, Keren Dunaway, Gnilane Turpin, Danielle Lonbong Njiometio, Uma Bhatt, Charity T. Mkona, Olena Stryzhak, Diana Weekes, Immaculate Owomugisha, Omar Syarif, Pim Looze, Jean De Dieu Anoubissi, Fletcher Chiu, Daria Ocheret, Laurel Sprague, Carlos Garcia De Leon Moreno, Stefan Baral, Global Scan Committee, Katherine Rucinski, Sophie Brion, Carrie Lyons

**Affiliations:** a Research Assistant, Johns Hopkins Bloomberg School of Public Health, Baltimore, USA. altheawolfe01@gmail.com; b Global Programme Officer, International Community of Women Living with HIV, Johannesburg, South Africa. k.dunaway@wlhiv.org; c Senior Research Manager, Johns Hopkins Bloomberg School of Public Health, Baltimore, USA. gturpin@jhu.edu; d Research Assistant, Johns Hopkins Bloomberg School of Public Health, Baltimore, USA. danielleln18@gmail.com; e Research Assistant, Johns Hopkins Bloomberg School of Public Health, Baltimore, USA. ubhatt1@alumni.jh.edu; f Chairperson, ICW Global and ICW Malawi, International Community of Women Living with HIV, Lilongwe, Malawi. charity.mkona@yahoo.com; g Positive Women Ukraine, Kyiv, Ukraine. elenas@ukr.net; h Regional Coordinator, ICW Caribbean, International Community of Women Living with HIV, Port of Spain, Trinidad and Tobago. dianeweekes360@gmail.com; i Attorney, Centre for Women Justice, Kampala, Uganda. iowomugishabazare@gmail.com; j Program Manager, Global Network of People Living with HIV, Amsterdam, The Netherlands. octoberomaro@gmail.com; k Senior Program Officer, Global Network of People Living with HIV, Amsterdam, The Netherlands. pimlooze@gmail.com; l Research Officer, Global Network of People Living with HIV, Amsterdam, The Netherlands. jd.anoubissi@gmail.com; m Program Officer, Global Network of People Living with HIV, Amsterdam, The Netherlands. fletcherchiu@gmail.com; n Community Led Responses, Advisor, UNAIDS, Geneva, Switzerland. matyushinada@unaids.org; o Chief, Community and Youth Engagement, UNAIDS, Geneva, Switzerland. laurelsprague@me.com; p Community and Youth Engagement, Greater Involvement of People Living with HIV (GIPA) Officer, UNAIDS, Geneva, Switzerland. garciadeleonc@unaids.org; q Professor, Johns Hopkins Bloomberg School of Public Health, Baltimore, USA. sbaral@jhu.edu; r Global Scan Committee, * International Community of Women Living with HIV, Johannesburg, South Africa. s.brion@wlhiv.org; s Associate Scientist, Johns Hopkins Bloomberg School of Public Health, Baltimore, USA. rucinski@jhu.edu; t Director of Global Programmes, International Community of Women Living with HIV, Johannesburg, South Africa. sophieicwglobal@gmail.com; u Research Associate, Johns Hopkins Bloomberg School of Public Health, Baltimore, USA. clyons8@jhmi.edu

**Keywords:** reproductive coercion, sexual and reproductive health, HIV, stigma, sterilisation, contraception, human rights

## Abstract

Biomedical advancements in HIV prevention and treatment have provided opportunities for women living with HIV to move through pregnancy, give birth, and breastfeed while effectively removing the risk of vertical transmission of HIV to their child. However, existing evidence suggests that the reproductive health and rights of women living with HIV are threatened through coercive practices in healthcare settings because of their HIV status. The objective of this scoping review was to synthesise evidence and identify gaps in the literature on reproductive coercion experienced by women living with HIV in clinical settings. This review focused specifically on reproductive coercion in the context of clinical healthcare globally, as opposed to intimate partnerships, and included forced or covertly performed sterilisation, forced abortion, restricted or forced contraceptive methods, forced or denied caesarean sections, and general coercion by healthcare providers regarding fertility-, sexual-, and reproductive-related decision-making. We searched three databases (Embase, PubMed, and LILACS) for quantitative, qualitative, and mixed methods studies. After 2888 unique publications were screened, thirteen publications met the inclusion criteria. Sterilisation was the most common coercion type assessed, and Mexico, the United States, and South Africa were common study settings. Variation in reproductive coercion definitions, study methods, and reporting was observed. Evidence from these studies suggests that reproductive coercion among women living with HIV is severe and pervasive. Therefore, there is a need to expand research on coercion and stigma that women living with HIV face while navigating reproductive healthcare. Additionally, prevention mechanisms and resource expansion for survivors beyond legal settings should be implemented globally.

## Plain language summary

Women living with HIV face barriers to quality healthcare, including stigma and discrimination. One barrier is reproductive coercion in healthcare settings. This broad term includes multiple types of human rights violations (including forced sterilisation and abortion) committed by healthcare providers on women seeking reproductive health services. To date, there has not been a formal synthesis of peer-reviewed research into reproductive coercion experienced by women living with HIV. We searched Embase, PubMed, and LILACS databases and screened over 2800 unique publications. The thirteen publications that met our search criteria spanned studies in Latin America, Sub-Saharan Africa, North America, and Southeast Asia. There were four main themes: 1. Common situations and types of healthcare visits where coercion takes place; 2. Power dynamics preventing women from making informed decisions about their reproductive health; 3. Negative health impacts of coercion; and 4. Prioritisation of preventing vertical HIV transmission over listening to women seeking care. This thematic analysis suggests that reproductive coercion among women living with HIV is varied, occurring globally, and greatly impacting women’s health and well-being.

## Résumé

Les progrès biomédicaux dans la prévention et le traitement du VIH ont permis aux femmes vivant avec le VIH de connaître la grossesse, l’accouchement et l’allaitement tout en éliminant efficacement le risque de transmission verticale du VIH à leur enfant. Néanmoins, les données existantes suggèrent que la santé et les droits reproductifs des femmes vivant avec le VIH sont menacés par des pratiques coercitives dans les établissements de santé en raison de leur statut sérologique. L’objectif de cette étude était de synthétiser les données et d’identifier les lacunes dans les publications sur la coercition reproductive subie en milieu clinique par les femmes vivant avec le VIH. L’étude s’est centrée plus spécialement sur la coercition reproductive dans le contexte des soins cliniques, par opposition aux relations intimes, et a inclus la stérilisation forcée ou pratiquée insidieusement, l’avortement forcé, les méthodes contraceptives restreintes ou forcées, les césariennes forcées ou refusées et la coercition générale exercée par les prestaires de soins de santé concernant les décisions en matière de fécondité, de sexualité et de procréation. Nous avons consulté trois bases de données (Embase, PubMed et LILACS) pour y chercher des études quantitatives, qualitatives et à méthodes mixtes. Après examen de 2888 publications uniques, treize publications répondaient aux critères d’inclusion. La stérilisation était le type de coercition le plus souvent évalué, et le Mexique, les États-Unis et l’Afrique du Sud étaient des sites fréquents d’étude. Des variations dans les définitions, les méthodes d’étude et les rapports relatifs à la coercition reproductive ont été observées. Les données tirées de ces études indiquent que la coercition reproductive chez les femmes vivant avec le VIH est grave et très répandue. Il est donc nécessaire d’élargir les recherches sur la coercition et la stigmatisation auxquelles font face les femmes vivant avec le VIH dans le cadre des soins de santé reproductive. De plus, des mécanismes de prévention et un renforcement des ressources pour les victimes au-delà des cadres juridiques devraient être mis en œuvre à l’échelle mondiale.

## Resumen

Los avances biomédicos en la prevención y el tratamiento del VIH han ofrecido oportunidades para que las mujeres que viven con VIH puedan llevar su embarazo a término, dar a luz y amamantar a la vez que eliminan eficazmente el riesgo de la transmisión vertical del VIH a su bebé. Sin embargo, la evidencia disponible indica que la salud y los derechos reproductivos de las mujeres que viven con VIH se ven amenazados por prácticas coercitivas en ámbitos de salud debido a su estado de VIH. El objetivo de esta revisión exploratoria era sintetizar la evidencia e identificar las brechas en la literatura sobre coerción reproductiva experimentada por mujeres que viven con VIH en entornos clínicos. Esta revisión se centró específicamente en la coerción reproductiva en el contexto de los servicios de atención clínica, a diferencia de parejas íntimas, y abarcó esterilización forzada o realizada de manera encubierta, aborto forzado, métodos anticonceptivos restringidos o forzados, cesáreas forzadas o denegadas y coerción general por prestadores de servicios de salud relativa a la toma de decisiones relacionadas con la fertilidad, la salud sexual y la salud reproductiva. Realizamos una búsqueda en tres bases de datos (Embase, PubMed y LILACS) de estudios cuantitativos, cualitativos y de métodos mixtos. De 2888 publicaciones únicas revisadas, trece publicaciones reunieron los criterios de inclusión. La esterilización fue el tipo de coerción más común evaluado; y México, Estados Unidos y Sudáfrica fueron los entornos comunes de los estudios. Se observó variación en las definiciones de coerción reproductiva, en los métodos de estudio y en los informes. La evidencia de estos estudios indica que la coerción reproductiva entre las mujeres que viven con VIH es grave y generalizada. Por ello, es necesario ampliar las investigaciones sobre la coerción y el estigma enfrentados por las mujeres que viven con VIH al navegar los servicios de salud reproductiva. Además, es imperativo aplicar a nivel mundial los mecanismos de prevención y ampliar los recursos para sobrevivientes más allá de los ámbitos jurídicos.

## Introduction

Sexual and reproductive healthcare has advanced considerably in recent decades, with technological, pharmacological, and clinical developments making both HIV and fertility treatments accessible for many women worldwide. Prevention of vertical transmission of HIV from mother to child is a global public health priority, with established interventions such as provision of antiretroviral therapy (ART) to women living with HIV both during and after pregnancy.^1^ These interventions, along with supportive structural and social interventions such as higher HIV testing coverage, have substantially reduced vertical transmission across settings.^1,2^ Globally, 72% of people living with HIV are virally suppressed,^3^ and among women who are virally suppressed, vertical transmission is fully preventable.^1,4^ With these advancements, along with widespread investment in programmatic interventions for the prevention of vertical transmission prioritised in settings across the world, there are safe opportunities for women living with HIV to pursue pregnancy and childbirth if they want to have children.^5,6^

Women living with HIV have a constellation of HIV, sexual and reproductive health, and primary healthcare needs. Studies conducted in recent years have shown that an HIV diagnosis does not decrease fertility desire, and that many women living with HIV who receive quality counselling want to become pregnant and have children.^7,8^ Sexual and reproductive health services are meant to support contraceptive needs, pregnancies, birth, and prevention of sexually transmitted infections (STIs) among all women, including women living with HIV.^9,10^ Women living with HIV have additional sexual and reproductive health needs due to elevated risk of STIs, human papillomavirus (HPV) and development of cervical cancer.^11^ Addressing these varied health needs via comprehensive sexual and reproductive health services is necessary for this population.^9,10,12–14^ However, access to these comprehensive services can be threatened by numerous social and environmental factors. Before women enter a clinic, they also face obstacles like intimate partner violence, male partner resistance or control over healthcare decisions, and economic barriers such as high transportation costs.^15^ Once in clinics, discrimination and misinformation regarding sexual and reproductive health and fertility options may represent obstacles for women living with HIV seeking healthcare services and can impact aspects of care such as disclosure of HIV status.^16^ Additionally, health-related stigma is a systemic and pervasive barrier that “enables *varieties of discrimination* that ultimately deny the individual/group *full social acceptance*, reduce the individuals’ opportunities, and fuel social inequalities.”^17,18^ HIV-related stigma is driven by social norm enforcement and fear of infection, facilitated by criminalising laws and poor health supply chains, and manifests in discrimination and experiences of poor healthcare.^17^

Amidst the health needs of women living with HIV, coercion and discrimination within healthcare facilities have the potential to lower the quality of care and expose women to human rights violations. Reproductive coercion by healthcare providers includes abusive and discriminatory practices that are designed to force reproductive decisions, including forced and coerced sterilisation, abortion, and contraceptive use, withholding informed consent, services, and counselling, and physical or verbal abuse towards women in childbirth (defined here and in other literature^19^ as obstetric violence).^20^ One survey conducted among women living with HIV across 94 countries found that 30% of respondents faced discrimination in healthcare settings, and women reported being forced to undergo sterilisation and abortion and use long-acting reversible contraceptive (LARC) methods.^21^ Women living with HIV are not the only population who experience reproductive coercion in clinical settings – historically and currently, women with diverse identities have reported reproductive coercion across many contexts. This has been studied in the context of eugenics and barriers to decision-making and control.^22,23^ Forced sterilisation in particular has been weaponised against many populations globally, including Black women in the American South, Jewish prisoners in the Holocaust, transgender people seeking legal transition, and other marginalised women such as migrants and disabled people.^23,24^ Contraceptive coercion more broadly is another area of research, and typically comprises “upward coercion” such as pressure to use specific methods, as well as “downward coercion” such as denial of contraceptive methods.^25^ A study in Western Kenya found that single, young women who did not have children were vulnerable to downward coercion, and that healthcare provider bias and outdated practices related to HIV were reported in relation to preferred method denial.^26^ Reproductive autonomy work has focused on areas of coercion such as pressuring women to use contraceptive implants without having infrastructure to guarantee removal of the device, lack of patient voice in decision-making, and postpartum pressure to use contraception.^22,26,27^

While these studies demonstrate that groups of marginalised women experience reproductive coercion while seeking healthcare, women living with HIV may experience both general types of reproductive coercion and coercion that is unique to the intersection of reproductive health and HIV status. In response, the objective of this scoping review was to synthesise evidence and identify gaps in the literature on reproductive coercion experienced by women living with HIV in clinical settings.

## Methods

We conducted a scoping review to identify and synthesise qualitative and quantitative evidence of experiences of reproductive coercion among women living with HIV. The search strategy aimed to identify studies that included women living with HIV and different types of reproductive coercion, using Medical Subject Headings (MeSH) and key terms. The full search protocol and strategies are provided in Supplementary File A. This scoping review followed the Preferred Reporting Items for Systematic reviews and Meta-Analyses extension for Scoping Reviews (PRISMA-ScR) methodology. A systematic search of a range of medical, public health, and social science research databases, including PubMed, Embase and LILACS, was conducted from March 2022 to February 2024. The search was restricted to papers published in a thirteen-year period (January 1st, 2011–January 26th, 2024) to obtain the latest overview of evidence. The HPTN 052 clinical trial results were published in 2011, marking a breakthrough in “treatment as prevention” and providing a strong foundation for all future research on early initiation of antiretroviral treatment to improve a variety of HIV-related outcomes.^28^ A scoping mixed studies review was chosen to best facilitate exploration and thematic analysis of many types of reproductive coercion experienced by women living with HIV across multiple contexts.

**Figure 1.**
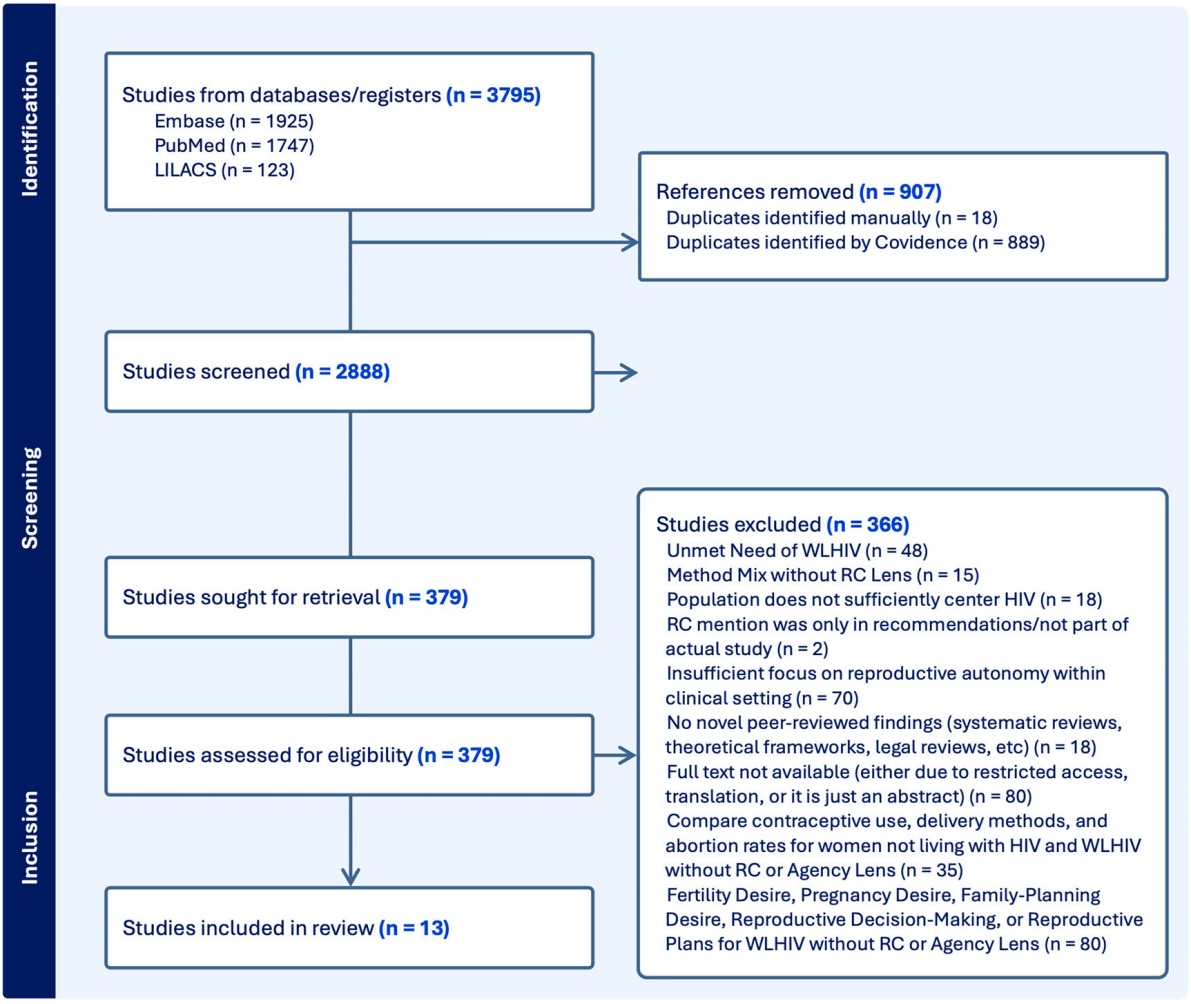
PRISMA flowchart detailing review process

An initial search was conducted for each database using the established search terms. Articles were then combined into the systematic review management system, Covidence.^29^ Duplicate articles were removed both automatically upon import and manually by screeners. Articles then underwent title/abstract screening and full-text review and were assessed for eligibility at each phase. Details of the search results by phase of the review are presented in the PRISMA flowchart (Figure 1).

Articles were eligible for inclusion if (1) the study focused on coercion experienced by women living with HIV in reproductive health settings, including both generally coercive experiences/indicators and specific areas of coercion, such as sterilisation, abortion, contraception, and pregnancy/birth practices; (2) the study method was either qualitative, quantitative, or mixed methods; and (3) the paper was written in English. No geographic limits were placed on the inclusion criteria, with the aim of identifying publications regardless of location. Abstracts were excluded for both technical reasons (if the article was published prior to January 1st, 2011 or not available in English) and content reasons (study population did not include women currently living with HIV, study’s only HIV-related component was focused on prevention, study was a clinical trial for pharmaceuticals/technologies, and study did not include sufficient public health/epidemiology focus (i.e. study was focused on HIV-related education in school systems)). The full-text articles were assessed independently by two screeners to obtain the final selection, with tiebreaking done by the first author. At the full-text stage, articles were excluded if the study was not focused on reproductive coercion, even if their objectives/design included family planning/HIV topics that have previously been connected to coercion. Studies excluded for this reason were tagged by topic, and the full list is provided in Figure 1. A grey literature database search was conducted after the independent screening had concluded, and any relevant articles were evaluated at the full-text stage by the first author.

A data-based convergent qualitative synthesis was utilised to structure the review, in which the quantitative, qualitative, and mixed methods data were transformed into qualitative themes and patterns. A range of commonly used extraction categories, such as year of study, country, study type, study population, key findings, and results, formed the initial extraction matrix, which was piloted in a single article and discussed by the review team. This matrix was compiled using Covidence and Excel.^29^ After the extraction matrix was finalised, charting was conducted for all thirteen articles included in the final group for review. After all the data were charted and compared for patterns among study methodologies, types of coercion explored, placement within reproductive health services, limitations and advantages, recommendations, and other indicators, the four main themes were selected. To ensure consistency and reliability of data synthesis, data analysis was conducted by the first author and independently reviewed by the remaining screeners and authors. Disparities were resolved by discussion and consensus among the authors. A formal quality assessment of the studies included in this review was conducted using the McGill Mixed Methods Appraisal Tool (MMAT) version 2018 (Supplementary File B).^30^

## Results

Overall, 3795 articles were identified through the initial search across databases, and 907 duplicates were removed. After de-duplication, 2888 articles underwent title and abstract screening, and 2509 were removed at this phase. Frequently cited removal reasons at the title and abstract screening included: the study population did not include any women currently living with HIV, and the study was a pharmaceutical-focused clinical trial. There were 379 articles that underwent full-text screening, of which 366 were determined to be ineligible. Reasons for ineligibility at full-text screening can be found in the PRISMA flow chart (Figure 1). Grey literature was searched via the World Health Organization database, but no articles were found that provided peer-reviewed or novel evidence-based findings that fit the goal of this review. Further explanation of full-text screening exclusion criteria can be found in Supplementary File C. Overall, 13 articles met all the eligibility criteria.

Article summaries are described in Table 1. Among the 13 articles, 3 were quantitative studies, 6 were qualitative studies, and 4 were mixed methods. MMAT quality assessment scores were generally high across study types, and reasons for lower scoring or negative comments varied by study type; while mixed methods studies scored highly and were noted to have high alignment across method-specific findings, qualitative studies often included very small sample sizes, and some quantitative studies were noted to lack information about exposure and comparison groups and confounding within the analytic strategy (Supplementary File B). Studies ranged geographically, with data from single-country studies coming from the United States (*n* = 3), Mexico (*n* = 2), South Africa (*n* = 2), Malaysia (*n* = 2), Brazil (*n* = 1), Namibia (*n* = 1), and Tanzania (*n* = 1). There was one multi-country study with data collection in Mexico, Honduras, El Salvador, and Nicaragua.

**Table 1.** Summary of publications on reproductive coercion experienced by women living with HIV identified and included in scoping review

Article	Country	Method	Study design	Population	Main finding
Bakare (2020)	Namibia	Qualitative	Interviews	7 WLHIV	Forced sterilisation has significant negative impacts on many aspects of mental health, physical health, and the ability to engage with community, and these impacts range from immediately post-sterilisation to long term
Barbosa (2016)	Brazil	Quantitative	Cross-sectional survey	975 WLHIV and 1003 WNLHIV	Living with HIV was associated with a higher probability of having a postpartum sterilisation and a lower probability of having an interval sterilisation
Cuca (2016)	USA	Qualitative	Interviews	20 WLHIV	Trauma and stigma had a significant impact on women's family planning decision-making and quality of care and manifested in abortion coercion for several participants
Kendall (2013)	Mexico	Qualitative	Interviews	55 in-depth interviews with WLHIV and 60 additional interviews with stakeholders	Contraceptive coercion was commonly reported in these interviews, with male condoms being pushed even when women told providers at HIV clinics that their partners would not be receptive to condom use or that they preferred different methods (sometimes in addition to condoms). Women also experienced other types of contraceptive coercion regarding IUDs and sterilisation, even when other methods were available
Kendall (2015)	El Salvador, Honduras, Mexico, Nicaragua	Mixed methods	Cross-sectional survey and interviews	285 Total WLHIV; El Salvador:56 Honduras:87 Mexico:82 Nicaragua:60	WLHIV were pressured to undergo sterilisation because of their HIV status in numerous ways, but many social and economic factors were found not to be related to sterilisation pressure. Women were most likely to experience pressure/coercion to sterilise when they found out their HIV status before their pregnancy or while pregnant. Coercion was also associated with younger ages. The findings from this study lay the foundation for establishing sterilisation coercion as a commonly reported and deeply harmful type of violence that is directly related to HIV status
Raziano (2017)	USA	Quantitative	Cross-sectional survey	187 WLHIV	Sterilisation regret was reported by women living with HIV, and many cited HIV status and pressure from others as key reasons they decided to undergo sterilisation. Additionally, among women who were sterilised, desire for future children was common
Rice (2019)	USA	Mixed methods	Cross-sectional survey and interviews	76 WLHIV interviews and 460 questionnaires	Anticipated and recently experienced stigma by healthcare providers were not reported with the same frequency, but both types of stigma were related to HIV adherence outcomes. Women also reported a lack of autonomy in interactions with healthcare providers
Strode (2012)	South Africa	Qualitative	Interviews	22 WLHIV	Women who had been forcibly or secretly sterilised reported discriminatory interactions with healthcare workers while seeking many different types of care, and often had their right to informed consent explicitly violated
Towriss (2019)	South Africa	Mixed methods	Longitudinal trial and interviews	471 WLHIV in a quantitative trial and 39 in-depth interviews	Women were heavily influenced to use injection methods instead of other available methods in the immediate postpartum period and had high rates of discontinuation and switching methods later in the postpartum period
van Dijk (2014)	Mexico	Qualitative	Interviews	31 Qualitative interviews	Women faced generally coercive environments with healthcare workers while trying to get fertility and pregnancy counselling
Sukeri (2024)	Malaysia	Qualitative	Interviews and focus group discussions	73 WLHIV; 11 in-depth interviews and 16 focus group discussions	The main themes among women living with HIV trying to make decisions about contraceptive use and future pregnancies were lack of negotiation, idealism in pregnancy, coping with restrictions, and past and future fears
Sando (2014)	Tanzania	Convergent parallel mixed methods	Interviews, cross-sectional survey, and direct observation	2000 Interviews with postpartum women, 208 direct childbirth observations, 50 women surveyed, and 18 in-depth interviews with healthcare providers	While HIV was not a risk factor for obstetric abuse and disrespect during childbirth, there were very high levels of abuse and disrespect among women observed and interviewed at the hospital facilities. This is a concern for all pregnant women, not just women living with HIV
Yadzir (2023)	Malaysia	Quantitative	Cross-sectional survey	141 WLHIV	Women reported coercion related to contraception (including informing women that they could only receive ART if they used contraception), infant feeding, and general stigma against having sex and getting pregnant

Types of reproductive coercion assessed were sterilisation only (*n* = 4); contraception and general coercion (*n* = 2); contraception only (*n* = 2); abortion and general coercion (*n* = 2); sterilisation and contraception (*n* = 1); sterilisation and general coercion (*n* = 1); and obstetric violence (*n* = 1).

Among the 13 articles, 10 were conducted in countries that have been highlighted in advocacy by the International Community of Women living with HIV (ICW).^20^ Additionally, three of the 13 articles included a Namibian involuntary sterilisation court case in their study rationale and objectives,^31–33^ and the study conducted in Namibia defined their target population as plaintiffs in this same case.^31^

The study populations and exposures of interest also varied greatly across these 13 articles. Among studies, three recruited women who had already experienced and reported forced or coerced sterilisation. For the other 10 studies, recruitment and data collection sources included women undergoing sterilisation procedures (*n* = 2), women seeking HIV-related services (*n* = 1), women living with HIV accessing fertility or family planning services (*n* = 6), and women giving birth in hospitals (*n* = 1).

Four themes emerged from the articles: (1) Coercion entry points throughout reproductive and pregnancy cycles (*n* = 11); (2) Power dynamics and decision-making (*n* = 9); (3) Health impacts and consequences of coercion (*n* = 6); and (4) Prioritisation of vertical transmission prevention over autonomy of women seeking care (*n* = 4). Thematic and structural characteristics of publications are described in Table 2.

**Table 2.** Thematic and structural characteristics of publications on reproductive coercion experienced by women living with HIV identified and included in scoping review

Article	Coercion method	Outcome	Exposure	Entry points	Power dynamic	Health impact	Prioritising others
Bakare (2020)	Sterilisation	Various psychosocial and physical health outcomes	Forced sterilisation and coercion to sterilise	Y	Y	Y	N
Barbosa (2016)	Sterilisation	Access to postpartum and interval sterilisation	HIV status, with further factors like number of children explored	Y	N	N	N
Cuca (2016)	Abortion and general coercion	Family planning choices and access, and experiences with healthcare workers	General pregnancy and fertility experiences in healthcare settings	Y	Y	Y	Y
Kendall (2013)	Contraception	Contraception choices and access, and experiences with healthcare workers	General pregnancy and fertility experiences in healthcare settings	Y	N	Y	N
Kendall (2015)	Sterilisation	Experiencing pressure to sterilise	Social and economic characteristics and fertility history	Y	Y	Y	Y
Raziano (2017)	Sterilisation and contraception	Sterilisation	Various social and clinical characteristics	N	N	Y	N
Rice (2019)	Sterilisation and general coercion	ART adherence	Anticipated and experienced stigma with healthcare worker interactions	N	Y	N	N
Strode (2012)	Sterilisation	Family planning choices and access, and experiences with healthcare workers	Sterilisation both forced/coerced and covert	Y	Y	Y	Y
Towriss (2019)	Contraception	Contraceptive method used immediately postpartum and 18 months postpartum	Contraceptive method initially chosen	Y	N	N	N
van Dijk (2014)	Abortion and general coercion	Contraception choices and access, and experiences with healthcare workers	General pregnancy and fertility experiences in healthcare settings	Y	Y	N	Y
Sukeri (2024)	Contraception and general coercion	Contraception choices and access, and experiences with healthcare workers	General pregnancy and fertility experiences in healthcare settings	Y	Y	N	N
Sando (2014)	Obstetric violence	Obstetric violence and abuse during childbirth	Living with HIV	Y	Y	N	N
Yadzir (2023)	Contraception and general coercion	Withholding of ART unless contraception initiated and baby formula fed, general counselling not to have sex or become pregnant	Contraceptive use	Y	Y	N	N

### Theme 1: Coercion entry points throughout reproductive and pregnancy cycles

**Figure 2.**
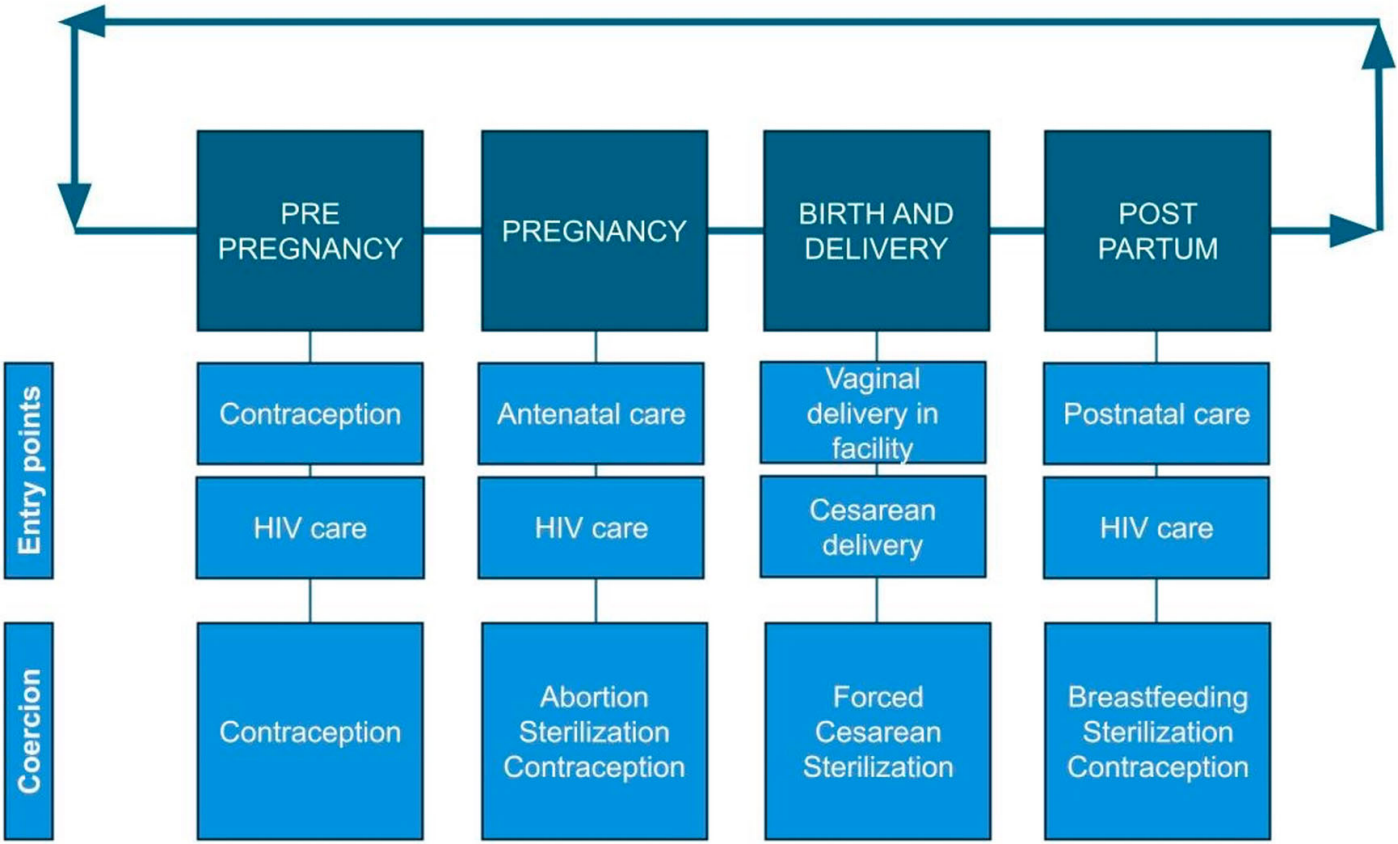
Opportunities for coercion at varying facility visits during the reproductive timeline

One theme that emerged was the existence of multiple entry points into healthcare in which a woman may be susceptible to coercive care or obstetric violence. These entry points exist along the cycle of pre-pregnancy, pregnancy, birth, and postpartum periods, and represent clinical visits that are opportunities for healthcare workers to force or coerce women into reproductive decisions (Figure 2). As 11 of the 13 total studies in this review displayed this theme, a description of each publication in the reproductive timeline by method of reproductive coercion is provided in Table 3. Women may be vulnerable to coercion during these entry points due to a change in healthcare provider or services sought to support a new or different reproductive health need.

**Table 3.** Situating each publication in the reproductive timeline by method of reproductive coercion

Article	Coercion method	Place in reproductive timeline
Bakare (2020)	Sterilisation	During labour and delivery
Barbosa (2016)	Sterilisation	During labour and delivery or separate from pregnancy (during an unspecified point in menstrual cycle)
Cuca (2016)	Abortion and general coercion	Before pregnancy (family planning) and making decision about continuing a pregnancy (abortion, which is typically coded as pregnancy-related care)
Kendall (2013)	Contraception	Before pregnancy and during the postpartum period (family planning)
Kendall (2015)	Sterilisation	All-sterilisation as contraceptive coercion after HIV diagnosis, threats of sterilisation after conclusion of current pregnancy, sterilisation during caesarean section, and sterilisation during unrelated surgery
Raziano (2017)	Sterilisation and contraception	Before pregnancy (family planning)
Rice (2019)	Sterilisation and general coercion	Separate from pregnancy (tubal ligation coercion during an unrelated healthcare visit for women who were not pregnant)
Strode (2012)	Sterilisation	During labour and delivery
Towriss (2019)	Contraception	Postpartum pregnancy prevention (at the very beginning of the next cycle of pregnancy care), including the immediate postpartum period
van Dijk (2014)	Abortion and general coercion	Before pregnancy (family planning) and during pregnancy
Sukeri (2024)	Contraception and general coercion	Before pregnancy and during postpartum period (family planning)
Sando (2014)	Obstetric violence	During labour and delivery
Yadzir (2023)	Contraception and general coercion	During pregnancy and postpartum period (family planning)

#### Before and during pregnancy

Seeking contraceptive or fertility counselling was an entry point for reproductive coercion explored in multiple studies. In a study in Mexico, women reported negative experiences with contraceptive counselling and withholding of non-condom methods.^34^ In studies conducted in the United States and Mexico, pregnant women seeking antenatal care experienced abortion coercion and significant shame and judgement from providers.^35,36^

#### During labour and delivery

Studies conducted across Latin America found that women living with HIV were forcibly sterilised or sterilised without their knowledge, typically during or immediately after giving birth.^32^ Women described the coercive conditions where providers withheld life-saving healthcare and time-sensitive resources, such as requested caesarean deliveries, formula for their newborn babies, and other types of treatment, until the women agreed to be sterilised.^32^ Most of the women interviewed in the South African study on forced, coerced and covert sterilisation believed the procedure was performed during a caesarean section or immediately after giving birth.^33^ All women in the study among survivors in the landmark Namibia sterilisation case were forcibly or secretly sterilised while having caesarean sections, as noted in the article.^31^ Among these seven women, four discovered they were secretly sterilised in the years after giving birth, and the other three were coerced to consent to sterilisation or discovered they had been sterilised in the immediate postpartum period.^31^ In the study in Mexico, women also described coercive tactics during labour or in pre-operation for caesarean sections, with consent forms for sterilisation presented repeatedly by providers despite refusals.^34^

Physical abuse and mistreatment were reported in a study in Tanzania, where women were observed during childbirth and interviewed about their delivery experiences.^37^ Rates of obstetric violence were high among all women regardless of HIV status.^37^

While the study conducted in Brazil did not highlight forced or coerced sterilisation, it explored differences in sterilisation access by timing of the procedure and by HIV status.^38^ The authors observed that for all types of postpartum sterilisation, women living with HIV with both 1–2 and 3 or more children had approximately two–three times the risk of being sterilised compared to women not living with HIV with the same number of children.^38^ Living with HIV was associated with a higher probability of having a postpartum sterilisation and a lower probability of having an interval sterilisation (performed any time other than postpartum).^38^ This points to a type of underlying contraceptive coercion in the form of only pursuing sterilisations for women living with HIV when performed in conjunction with caesarean sections. Moreover, it lays the foundation for sterilisation coercion during childbirth or the immediate postpartum period, when women may be in distress.

#### Postpartum

In South Africa, 74% of postpartum women used injectable contraception immediately after birth.^39^ However, as the postpartum period continued, there were high levels of discontinuation, with 21% of the full study population not using any contraception at 18 months postpartum.^39^ This change was largely attributed to discontinuation of injectables, while both the use of other methods and childbearing intentions remained consistent across time.^39^ Additionally, the methods used immediately postpartum were also inconsistent with the methods preferred at the baseline antenatal care visit, and the majority of women who were interested in IUDs and contraceptive pills were using injectables one week postpartum.^39^

In the two studies conducted in Malaysia, women reported coercion by providers to use implants or undergo tubal ligation,^40^ and coercive counselling on infant feeding and contraceptive uptake requirements.^41^ In Mexico, women experienced similar postpartum coercion to use specific contraceptive methods, with many describing coercive environments where they had to pick an IUD or sterilisation or face shaming and anger from their healthcare provider.^34^ None of the women in this Mexican study reported forced sterilisation during childbirth, but the delivery and immediate postpartum period were marked by coercive tactics to undergo sterilisation or LARC uptake.^34^

### Theme 2: Power dynamics and decision-making

A second theme was the power dynamic between clinicians and women seeking care. This manifested in several ways: decision-making in the context of misinformation, withholding other types of care, and using positioning in a hospital or clinic to perform invasive procedures without consent.

Violation of autonomy in decision-making was a theme for women who had been forcibly or secretly sterilised in Namibia,^31^ Latin America^32^ and South Africa.^33^ In Namibia, healthcare providers made the decision to sterilise women because of their HIV status, and this decision clashed with other cultural factors that put decision-making in the custody of the women’s husbands, families and elders.^31^ Women in Namibia reported in interviews that their marriages suffered because decision-making power was taken away from their husbands, who culturally should have the final say in their medical choices, and reacted with verbal and emotional abuse.^31^ This impact on their relationships played into stereotypes about childbearing expectations, and sometimes led to rejection by their community support systems.^31^

In Honduras, Nicaragua, El Salvador and Mexico, women were lied to or given outdated information about sexual and reproductive options, which convinced many of them to consent to sterilisation they did not want.^32^ In South Africa, women reported similar withholding of information about risks of HIV and pregnancy, including providers telling them that the procedure was easily reversible or non-surgical.^33^ Another study in the United States found that the HIV-related stigma that women living with HIV anticipated they would encounter while seeking healthcare was higher than the stigma they reported experiencing in healthcare settings.^42^ However, women in this study did report pressure to sterilise and other types of discriminatory and coercive behaviour by their providers.^42^

In the United States-based study on fertility desire and general reproductive coercion, women living with HIV encountered judgement and shame connected to religious beliefs. One woman was told “*if God was here, he’d probably strike you to hell already”* by a nurse practitioner at her first appointment after becoming pregnant.^35^ Women in multiple studies in the United States and Mexico who experienced abortion coercion said that their HIV diagnosis was highlighted as the reason they wouldn’t be a good mother, and the manifestation of this stigma was low-quality or non-existent pregnancy care.^35,36^ One woman had an adverse birth outcome that was a direct result of doctors not listening to her during labour, which she reported was due to HIV stigma.^35^

In the hospital-based study on obstetric violence and mistreatment in Tanzania, HIV was not found to be a risk factor for most types of obstetric abuse, but nonconsensual first examinations and vaginal examinations in the antenatal ward were more commonly performed on women living with HIV compared to women not living with HIV.^37^ In a study conducted in government-controlled facilities in Malaysia, ART was withheld from postpartum women unless they used contraception and committed to formula-feeding their infant.^41^

### Theme 3: Health impacts and consequences of coercion

There were many physical, psychological and interpersonal impacts in the short- and long-term periods following sterilisation. Women who were involuntarily sterilised in Namibia reported a range of physical side effects, including long-term heavy bleeding that required them to wear diapers.^31^ These same women also reported negative impacts on their emotional and financial well-being, along with adverse mental health outcomes such as depression and anxiety, feelings of shame and worthlessness, and isolation.^31^ In other settings, women also reported feelings of shame and disgust after interactions with healthcare providers due to being diagnosed with HIV or being pregnant and living with HIV.^35^ Many participants reported self-isolation because of these negative healthcare interactions, highlighting how reproductive coercion may perpetuate internalised HIV-related stigma and impact relationships and community for women. In the United States, a study on factors associated with sterilisation found that sterilisation regret was common, and pointed towards coercion, incomplete counselling, or pressure from others that may have been undetected during the initial clinical visits.^43^ In all studies focusing on forced and coerced sterilisation, women reported compounding harm to their financial, physical, and mental health that was made worse by cultural “damage” and loss of community support over time.^31–33^

Women living with HIV reported only being provided condoms as a method of contraception. This reduction of available contraceptive options to women living with HIV impacted relationship dynamics and power during reproductive negotiation at home. Women in Mexico reported that their partners reacted with rejection, anger, accusations of infidelity, and sometimes physical violence when asked to use a condom during sex.^34^ Provider restriction of covert contraceptive methods that are controlled by women exposed them to violence and introduced dependence on male partners.^34^

### Theme 4: Prioritisation of vertical transmission prevention over autonomy of women seeking care

Women believed that some of the blame and negative reactions they received from providers when they told them about fertility desires were due to the perceived risk of vertical transmission of HIV. In the United States, there were reports of providers having told women living with HIV that they were selfish if they wanted to become pregnant, because of HIV, poverty and other aspects of medical history shortening life expectancy.^35^ In Mexico, although all women in the study continued with pregnancies, many reported pressure to terminate because of the potential risk to the baby.^36^ This was typically paired with shaming or derogatory remarks from providers about their decision to “*bring children with AIDS to the world.*”^36^ Some women even reported that providers who made similar remarks also refused to enrol them in the vertical transmission programme.^36^

Women in Latin America were concerningly called “*vectors of HIV disease,*” a stigmatising sentiment by providers who were coercing them to agree to sterilisation.^32^ Many of these women were also told that their children would die or that they would die during pregnancy or childbirth, and that sterilisation was the only way to prevent this from happening.^32^ Women who had been diagnosed with HIV during pregnancy often did not have adequate knowledge about ART options and vertical transmission risks, and could not counter these provider claims.

## Discussion

The thirteen studies included in the review cover a wide range of coercion types and geographic regions, but generally, few studies have empirically examined reproductive coercion among women living with HIV. Among the peer-reviewed articles identified in this review, there is a consistent reference to human rights instruments, legal protections, and advocacy. Much of the legal advocacy documentation has focused on forced and coerced sterilisation: this includes claims litigated against the governments of Chile, Kenya, Namibia, and others, with the case in Namibia escalating to the Supreme Court.^20^ In 2022, a court in Kenya encouragingly deemed sterilisation of women living with HIV without consent to be a violation of the right to dignity, freedom from discrimination, and the highest standard of health and reproductive freedom.^44^ HIV-related sterilisation in the twenty-first century has gained more mainstream attention through this legal advocacy, which is reflected in the inclusion of landmark cases in many of the studies in this review. From civil suit plaintiffs comprising the target population of qualitative research,^31^ to the findings of the court directly inspiring the research question,^32^ the history of HIV-related reproductive coercion in legal spaces informs context and current positioning in epidemiology. In summary, this scoping review highlights that few studies have assessed reproductive coercion among women living with HIV thus far and that formal research has apparently lagged behind numerous legal reviews and court opinions.^45–48^

Women living with HIV face multiple types of reproductive coercion, including those specific to HIV treatment opportunities and stigma.^48^ Evidence from our review suggests that these coercive practices can be connected to misinformation about the vertical transmission risk of HIV. Additionally, women reported experiencing stigma related to their HIV status, with mistreatment relating to their perceived ability to parent and to the degree they “deserve” motherhood. HIV-related stigma has persisted alongside critical events in HIV prevention, treatment, advocacy, and community throughout the last decade, which have built on early breakthroughs in treatment like the 1996 development of the HAART protocol.^49^ More recent events include the development and socialisation of early ART initiation protocols in 2011, laying the groundwork for U = U guidelines and advocacy,^28^ FDA approval of pre-exposure prophylaxis (PrEP) in 2012,^50^ and the current innovations in long-acting injectable PrEP.^51,52^ Despite these biomedical advancements making significant differences in HIV-related outcomes, HIV-related stigma is still embedded in modern-day healthcare structures. Today, reproductive coercion experienced by women living with HIV can include forced and pressured procedures such as sterilisations, abortions, uptake of specific contraceptives and caesarean sections.^20,21^ Behaviours such as withholding informed consent and refusing to provide accurate or quality obstetric care, fertility counselling, and contraceptive services are also included under this umbrella.^20,21^ The postpartum period is a particularly vulnerable time, marked by recovery, adjustment to life with a newborn, financial and relationship stressors, and numerous mental health risks. Direct assessments of coercion experienced by postpartum women living with HIV are valuable, but even indirect assessments (as demonstrated in the study on postpartum contraceptive use in South Africa) add to the existing knowledge base.^39^ The patterns in method-specific contraceptive use and discontinuation provide information about the postpartum entry point, highlighting techniques through which coercive interactions in clinical settings could be discovered, and contextualising how women living with HIV may engage with reproductive healthcare going forward.^39^ This review highlights that reproductive coercion takes many forms, and understanding the full body of evidence for women living with HIV can inform further research and interventions. Variation in origin and the heterogeneity of definitions, exposures, and outcomes make it challenging to measure in any standardised methodology.

Women living with HIV access reproductive healthcare all over the world, and reproductive coercion has been reported and studied in many different geographic contexts.^21^ The body of evidence evaluated in this review came from low-, middle-, and high-income countries across Latin America, Sub-Saharan Africa, East Asia, and North America. Family planning norms that connect to gender structures and cultural practices influence family planning discrepancies between women and their healthcare providers, along with the ramifications of forced sterilisation. One of the studies conducted in Malaysia referenced “pigeon pair” children as the “pinnacle” of childbearing, giving cultural context as women who had yet to give birth to both a boy and a girl were incentivised towards another pregnancy.^40^ The cultural drivers of ideal family sizes, contraceptive trends and HIV-related stigma appear to vary regionally and nationally. In addition to the high uptake of short-acting reversible contraceptive methods such as oral contraceptive pills and injectables^53,54^, female sterilisation is regionally the most commonly used method in Latin America and the Caribbean broadly.^55^ Given sterilisation is a common form of contraception in this region, these patterns may mask coercion and forced procedures contributing to increased use among women living with HIV specifically. Examining coercion experienced by women living with HIV seeking antenatal and postpartum care separately from women seeking family planning services may provide insight and clarification of context-specific coercion. This is distinct from the contexts of South Africa and Namibia, where influences include legal advocacy,^46,47^ HIV stigma,^56^ and regional popularity of implants and injectable contraception.^54^ The global nature of this review also highlights the need for coercion prevention, reporting mechanisms and clinical treatment scale-up to be integrated in all regions, not just countries with well-known civil suits.

Integration of HIV and sexual and reproductive health services, such as family planning, was recommended directly by several included articles as a potentially impactful prevention mechanism, including at the facility/service level^34,39^ and within provider training,^32^ and indirectly as a component of broader quality of care overhauls.^37^ Stigma from reproductive health practitioners regarding HIV transmission and available treatment options was pervasive. Conversely, exclusion of reproductive care and fertility counselling at HIV clinics limited women of reproductive age to HIV-related care only, leaving women to navigate separate clinical systems with more opportunity for misinformed or stigma-based counselling. “One stop shops” where these two types of healthcare are skilfully integrated have been explored in research: one longitudinal study in Zambia found that among women who reported pregnancy, the percentage who wanted the pregnancy doubled, increasing from 35% to almost 71% with the integration intervention.^57^ Other studies have leveraged qualitative interviews to explore considerations for HIV and sexual and reproductive health service integration, with one study in Kenya finding mistiming of contraceptive method and ART appointments to be a commonly reported obstacle.^58^ Having thoughtfully integrated, comprehensive and standardised facility-based care designed to minimise this burden on women living with HIV could also address related health disparities and improve population-level outcomes like dual contraceptive use, unmet need and demand satisfied.^57^ Unmet need among populations of women living with HIV has been characterised as both high and dynamically changing.^59^ Women living with HIV often also face elevated rates of unplanned pregnancy.^60^ As this review tracked foundational family planning indicators adjacent to coercion (such as unmet need and method mix), it found the number of articles on these topics was far greater than the number looking at coercion in the context of human rights violations.

Several limitations should be considered in the interpretation of these findings. First, although legal and political advocacy is crucial in understanding the evolution of reproductive coercion as a human rights violation and an urgent area for healthcare delivery improvement, the format of this scoping review of peer-reviewed literature did not allow for other types of literature, including legal advocacy publications, to be analysed. HIV-specific reproductive coercion has been documented as a human rights violation in multiple national court systems and globally by groups such as the International Community of Women living with HIV.^20^ Also, while we did include LILACS in our database search to broaden results in Latin America, our restriction to articles published in English may have resulted in the under-representation of some regions. Additionally, the grey literature search was restricted to a single search of the World Health Organization database. While this strategy did not yield any articles that matched the review criteria, the literature found in the search informed the introduction and discussion sections. Finally, heterogeneity of study designs and statistical analysis prevented us from including a quantitative meta-analysis as a part of the scoping review.

## Conclusion

Reproductive coercion is both a public health and human rights concern, but is often incorrectly presented as a narrowly defined and rare type of rights violation, separate from healthcare provider quality and separate from any prevention mechanism. Understanding the varied and nuanced coercive actions that have been reported in a wide variety of healthcare settings can help raise awareness of and address the harm done to many women living with HIV across the world. Validated measures and standardised indicators related to reproductive coercion among women living with HIV may facilitate better evaluation of the prevalence, social determinants, and impact of reproductive coercion over the life course. Seamless integration of HIV and sexual and reproductive health, both in healthcare delivery and in research, could catalyse the development of targeted prevention and reporting approaches specific to the previously explored entry points for coercion. Further analysis on the relationship between reproductive healthcare quality for women living with HIV and country-specific beliefs surrounding HIV and pregnancy could help further describe the context in which coercion and abuse take place. Incorporating reproductive coercion prevention and awareness in medical and research settings, not just legal settings, may reduce the overall mental, physical and emotional toll on women living with HIV seeking reproductive health services.

## Supplementary Material

Supplementary File B. Quality Evaluation Using the Mixed Methods Appraisal Tool (MMAT), Version

Supplementary File A. Search strategy.

Supplementary File C. RC Scoping Review Full-Text Exclusion Criteria.
