# Supplementary material for: Reproductive coercion experienced by women living with HIV – a global scoping review
Source: Sex Reprod Health Matters. 2026 Feb 3;33(1):2588004. doi: 10.1080/26410397.2025.2588004 (PMC12954806; doi:10.1080/26410397.2025.2588004)
Supplement: Supplementary File B. Quality Evaluation Using the Mixed Methods Appraisal Tool (MMAT), Version [file ZRHM_A_2588004_SM5009.docx]

# Supplementary File B. Quality Evaluation Using the Mixed Methods Appraisal Tool (MMAT), Version 2018

# Citation List:

# Bakare K, Gentz S. Experiences of forced sterilisation and coercion to sterilise among women living with HIV (WLHIV) in Namibia: an analysis of the psychological and socio-cultural effects. Sex Reprod Health Matters. 2020 Dec;28(1):1758439. doi: 10.1080/26410397.2020.1758439. PMID: 32436814; PMCID: PMC7887903.

# Barbosa RM, Cabral CD, do Lago TD, Pinho AA. Differences in the Access to Sterilization between Women Living and Not Living with HIV: Results from the GENIH Study, Brazil. PLoS One. 2016 Nov 3;11(11):e0164887. doi: 10.1371/journal.pone.0164887. PMID: 27812146; PMCID: PMC5094764.

# Cuca YP, Rose CD. Social Stigma and Childbearing for Women Living With HIV/AIDS. Qualitative Health Research. 2016;26(11):1508-1518. doi:10.1177/1049732315596150

# Kendall T. Falling short of universal access to reproductive health: unintended pregnancy and contraceptive use among Mexican women with HIV. Cult Health Sex. 2013;15 Suppl 2:S166-79. doi: 10.1080/13691058.2013.798685. Epub 2013 Jun 20. PMID: 23782295.

# Kendall T, Albert C. Experiences of coercion to sterilize and forced sterilization among women living with HIV in Latin America. J Int AIDS Soc. 2015 Mar 24;18(1):19462. doi: 10.7448/IAS.18.1.19462. PMID: 25808633; PMCID: PMC4374084.

# Raziano VT, Smoots AN, Haddad LB, Wall KM. Factors associated with sterilization among HIV-positive US women in an urban outpatient clinic. AIDS Care. 2017 May;29(5):612-617. doi: 10.1080/09540121.2016.1255710. Epub 2016 Dec 4. PMID: 27915483.

# Rice WS, Turan B, Fletcher FE, Nápoles TM, Walcott M, Batchelder A, Kempf MC, Konkle-Parker DJ, Wilson TE, Tien PC, Wingood GM, Neilands TB, Johnson MO, Weiser SD, Turan JM. A Mixed Methods Study of Anticipated and Experienced Stigma in Health Care Settings Among Women Living with HIV in the United States. AIDS Patient Care STDS. 2019 Apr;33(4):184-195. doi: 10.1089/apc.2018.0282. PMID: 30932700; PMCID: PMC6459270.

# Strode A, Mthembu S, Essack Z. "She made up a choice for me": 22 HIV-positive women's experiences of involuntary sterilization in two South African provinces. Reprod Health Matters. 2012 Dec;20(39 Suppl):61-9. doi: 10.1016/S0968-8080(12)39643-2. PMID: 23177681.

# Towriss CA, Phillips TK, Brittain K, Zerbe A, Abrams EJ, Myer L. The injection or the injection? Restricted contraceptive choices among women living with HIV. Sex Reprod Health Matters. 2019 Dec;27(1):1628593. doi: 10.1080/26410397.2019.1628593. PMID: 31533578; PMCID: PMC6754114.

# van Dijk, Marieke G. MA, MD; Wilson, Kate S. MPH; Silva, Martha MPH; Contreras, Xipatl BA; Fukuda, Dawn H. ScM; García, Sandra G. ScD. Health Care Experiences of HIV-Infected Women With Fertility Desires in Mexico: A Qualitative Study. Journal of the Association of Nurses in AIDS Care 25(3):p 224-232, May 2014. | DOI: 10.1016/j.jana.2013.04.006

# Sando D, Kendall T, Lyatuu G, Ratcliffe H, McDonald K, Mwanyika-Sando M, Emil F, Chalamilla G, Langer A. Disrespect and abuse during childbirth in Tanzania: are women living with HIV more vulnerable? J Acquir Immune Defic Syndr. 2014 Dec 1;67 Suppl 4(Suppl 4):S228-34. doi: 10.1097/QAI.0000000000000378. PMID: 25436822; PMCID: PMC4251905.

# Sukeri S, Sulaiman Z, Hamid NA, Ibrahim SA. Decision-Making on Contraceptive Use among Women Living with Human Immunodeficiency Virus in Malaysia: A Qualitative Inquiry. Korean J Fam Med. 2024 Jan;45(1):27-36. doi: 10.4082/kjfm.23.0088. Epub 2023 Oct 18. PMID: 37848368; PMCID: PMC10822730.

# Yadzir ZHM, Ramly M, Suleiman A. Do women living with human immunodeficiency virus experience stigma and discrimination when accessing sexual and reproductive health services in government health facilities in Malaysia? J Health Manag. 2023;20(1):96-101

#

# Article Citation: Bakare K, Gentz S. Experiences of forced sterilisation and coercion to sterilise among women living with HIV (WLHIV) in Namibia: an analysis of the psychological and socio-cultural effects. Sex Reprod Health Matters. 2020 Dec;28(1):1758439. doi: 10.1080/26410397.2020.1758439. PMID: 32436814; PMCID: PMC7887903.

| **Category of study designs** | **Methodological quality criteria** | **Responses** | | |
| --- | --- | --- | --- | --- |
|  |  | Yes | No | Can’t tell |
| Screening questions (for all types) | S1. Are there clear research questions? | X |  |  |
|  | S2. Do the collected data allow to address the research questions? | X |  |  |
|  | *Further appraisal may not be feasible or appropriate when the answer is ‘No’ or ‘Can’t tell’ to one or both screening questions.* | | | |
| 1. Qualitative | 1.1. Is the qualitative approach appropriate to answer the research question? | X |  |  |
|  | 1.2. Are the qualitative data collection methods adequate to address the research question? | X |  |  |
|  | 1.3. Are the findings adequately derived from the data? | X |  |  |
|  | 1.4. Is the interpretation of results sufficiently substantiated by data? | X |  |  |
|  | 1.5. Is there coherence between qualitative data sources, collection, analysis and interpretation? | X |  |  |

**Bakare 2020 Comments:**

1. The study aimed to explore individuals’ perceptions and their understanding around forced and coerced sterilization. Qualitative approaches are logical and appropriate for research questions on human rights violations, stigma and discrimination, and health equity.
2. The target population was small, and semi-structured interviews were adequate for the small number of participants and due to the sensitivity of the topic of the research question. Conducting semi-structured interviews allowed participants to be more open and willing to share their experiences and perceptions around forced and coerced sterilization. The decision to supplement the interviews with a questionnaire helped to reach saturation and pull novel themes from data collected through interviews.
3. The themes and quotes comprising the findings are relevant to the questions asked during data collection. The mental, emotional, financial, interpersonal, and physical impacts of forced and coerced sterilization were explored thoroughly through the interview guide. None of the themes, highlighted quotes, or other specific findings were irrelevant or outside the bounds of the qualitative inquiry.
4. The interpretation of these results was substantiated by the data. The recommendations also highlighted the need to address psychological symptoms like mental health problems that might emerge after coerced or forced sterilization.
5. The needs and experiences of the small target population, women living with HIV reporting forced or coerced sterilization in a civil suit, was clearly addressed throughout the study. From the decision to use a qualitative methodology for rich data, to the through thematic analysis, to the interpretation, there was coherence.

# Article Citation: Barbosa RM, Cabral CD, do Lago TD, Pinho AA. Differences in the Access to Sterilization between Women Living and Not Living with HIV: Results from the GENIH Study, Brazil. PLoS One. 2016 Nov 3;11(11):e0164887. doi: 10.1371/journal.pone.0164887. PMID: 27812146; PMCID: PMC5094764.

| **Category of study designs** | **Methodological quality criteria** | **Responses** | | |
| --- | --- | --- | --- | --- |
|  |  | Yes | No | Can’t tell |
| Screening questions (for all types) | S1. Are there clear research questions? | X |  |  |
|  | S2. Do the collected data allow to address the research questions? | X |  |  |
|  | *Further appraisal may not be feasible or appropriate when the answer is ‘No’ or ‘Can’t tell’ to one or both screening questions.* | | | |
| 3. Quantitative non-randomized | 3.1. Are the participants representative of the target population? | X |  |  |
|  | 3.2. Are measurements appropriate regarding both the outcome and intervention (or exposure)? | X |  |  |
|  | 3.3. Are there complete outcome data? | X |  |  |
|  | 3.4. Are the confounders accounted for in the design and analysis? | X |  |  |
|  | 3.5. During the study period, is the intervention administered (or exposure occurred) as intended? | X |  |  |

**Barbosa 2016 Comments:**

1. The study evaluated the impact of HIV-status on women’s access to different types of sterilization in Brazil. The recruitment methodology ensured that the study participants were representative of the target population.
2. The participants included women living with HIV and women not living with HIV, and both groups had diversity in parity, types of sterilization, and other demographic information.
3. The manuscript includes statistical data on the outcome, with multiple models available for different sub-questions. Complete outcome data is also shared through tables (which account for missing data) and figures.
4. Models for data analysis were adjusted for schooling, race, and parity at the last delivery. Data collection of these variables was also integrated into the study design.
5. The cross-sectional study format lead to the exposure occurring in the past or as an ongoing demographic factor for the participants. This was appropriate for the study design.

# Article Citation: Cuca YP, Rose CD. Social Stigma and Childbearing for Women Living With HIV/AIDS. Qualitative Health Research. 2016;26(11):1508-1518. doi:10.1177/1049732315596150

| **Category of study designs** | **Methodological quality criteria** | **Responses** | | |
| --- | --- | --- | --- | --- |
|  |  | Yes | No | Can’t tell |
| Screening questions (for all types) | S1. Are there clear research questions? | X |  |  |
|  | S2. Do the collected data allow to address the research questions? | X |  |  |
|  | *Further appraisal may not be feasible or appropriate when the answer is ‘No’ or ‘Can’t tell’ to one or both screening questions.* | | | |
| 1. Qualitative | 1.1. Is the qualitative approach appropriate to answer the research question? | X |  |  |
|  | 1.2. Are the qualitative data collection methods adequate to address the research question? | X |  |  |
|  | 1.3. Are the findings adequately derived from the data? | X |  |  |
|  | 1.4. Is the interpretation of results sufficiently substantiated by data? | X |  |  |
|  | 1.5. Is there coherence between qualitative data sources, collection, analysis and interpretation? | X |  |  |

**Cuca 2016 Comments:**

1. This study aimed to explore the experiences and reproductive decision-making of women living with HIV within situations limiting their ability to make truly informed choices. It used a qualitative approach (in-depth interviews and participant observation) to develop theories based on “sensitizing concepts” observed in the data and examine the participants' experiences and situations.
2. Data was collected mainly through in-depth interviews. This allowed participants to share experiences of discrimination and the situations that drive their reproductive decision-making process.
3. Approaches like open coding of the data helped identify drivers, barriers, and systemic obstacles within the reproductive decision-making process of women living with HIV. Ideas and themes noted from the interviews were also categorized into concepts or themes, such as trauma.
4. The discussion is supported by themes and quotes from the results (obtained after data coding and analysis).
5. The study population, data collection methods, quotes and themes, and interpretation of findings all connect to the research question on reproductive decision-making.

**Article Citation: Kendall T. Falling short of universal access to reproductive health: unintended pregnancy and contraceptive use among Mexican women with HIV. Cult Health Sex. 2013;15 Suppl 2:S166-79. doi: 10.1080/13691058.2013.798685. Epub 2013 Jun 20. PMID: 23782295.**

| **Category of study designs** | **Methodological quality criteria** | **Responses** | | |
| --- | --- | --- | --- | --- |
|  |  | Yes | No | Can’t tell |
| Screening questions (for all types) | S1. Are there clear research questions? | X |  |  |
|  | S2. Do the collected data allow to address the research questions? | X |  |  |
|  | *Further appraisal may not be feasible or appropriate when the answer is ‘No’ or ‘Can’t tell’ to one or both screening questions.* | | | |
| 1. Qualitative | 1.1. Is the qualitative approach appropriate to answer the research question? | X |  |  |
|  | 1.2. Are the qualitative data collection methods adequate to address the research question? | X |  |  |
|  | 1.3. Are the findings adequately derived from the data? | X |  |  |
|  | 1.4. Is the interpretation of results sufficiently substantiated by data? | X |  |  |
|  | 1.5. Is there coherence between qualitative data sources, collection, analysis and interpretation? | X |  |  |

**Kendall 2013 Comments:**

1. This study aims to provide a deeper understanding of the factors that shape reproductive decision-making and contraceptive use for women living with HIV in Mexico and their experiences with reproductive healthcare services and providers through in-depth interviews. A qualitative approach is appropriate to focus on the depth and breadth of those heterogenous experiences.
2. In-depth interviews are adequate to gain insights in the research question (the reproductive trajectories of Mexican women with HIV and their access and use of contraceptives). The short survey is complementary to the interviews, providing descriptive statistics about participants, and is also helpful for data collection and discussion.
3. The results include themes, quotes, and the excerpt from one of the interviews. These are also relevant to the research question.
4. The discussion highlights how cultural, social, and economic factors contribute to inconsistent condom use (one of the results from the data). In addition, one of the recommendations on scaling-up careful supervision to ensure that women with HIV are not coerced into accepting a particular contraceptive method is relevant to the research question.
5. The study population, data collection methods, quotes and themes, and interpretation of findings all connect to the research question on reproductive and family planning decision-making by women living with HIV in Mexico

# Article Citation: Kendall T, Albert C. Experiences of coercion to sterilize and forced sterilization among women living with HIV in Latin America. J Int AIDS Soc. 2015 Mar 24;18(1):19462. doi: 10.7448/IAS.18.1.19462. PMID: 25808633; PMCID: PMC4374084.

| **Category of study designs** | **Methodological quality criteria** | **Responses** | | |
| --- | --- | --- | --- | --- |
|  |  | Yes | No | Can’t tell |
| Screening questions (for all types) | S1. Are there clear research questions? | X |  |  |
|  | S2. Do the collected data allow to address the research questions? | X |  |  |
|  | *Further appraisal may not be feasible or appropriate when the answer is ‘No’ or ‘Can’t tell’ to one or both screening questions.* | | | |
| 5. Mixed methods | 5.1. Is there an adequate rationale for using a mixed methods design to address the research question? | X |  |  |
|  | 5.2. Are the different components of the study effectively integrated to answer the research question? | X |  |  |
|  | 5.3. Are the outputs of the integration of qualitative and quantitative components adequately interpreted? | X |  |  |
|  | 5.4. Are divergences and inconsistencies between quantitative and qualitative results adequately addressed? | X |  |  |
|  | 5.5. Do the different components of the study adhere to the quality criteria of each tradition of the methods involved? | X |  |  |

**Kendall 2015 Comments:**

1. The research goal is to assess what other characteristics other than HIV may contribute to healthcare providers engaging in coercive sterilization. The qualitative approach explores accounts of women pressured to undergo sterilization by providers, and the quantitative approach analyzes associations between characteristics and women’s experience of forced sterilization by providers. Both methods are appropriate for the aspect of the research question they help answer.
2. The questionnaire consisted of multiple-choice and open-text questions that were asked of participants. The open-text questions helped obtain details about women living with HIV’s experiences of reproductive rights violations, and the multiple-choice questions were focused on characteristics that may have contributed to reproductive rights violations from healthcare providers. Quantitative and qualitative components were integrated into the data collection instrument (questionnaire) to study both aspects of the research question.
3. The results are presented as tables, quotes, and themes relevant to the research question. Each type of result is presented adequately: themes and quotes for qualitative results and percentages and odds ratios for quantitative results. In addition, the discussion highlights both types of results.
4. The study did not share any inconsistencies between qualitative and quantitative results. Both types of results appear to be complementary. For example, younger participants had higher odds (quantitative results) of being pressured to be sterilized and were among the group with more reports of forced sterilization.
5. Both components of the study adhere to the appropriate quality criteria.

# Article Citation: Raziano VT, Smoots AN, Haddad LB, Wall KM. Factors associated with sterilization among HIV-positive US women in an urban outpatient clinic. AIDS Care. 2017 May;29(5):612-617. doi: 10.1080/09540121.2016.1255710. Epub 2016 Dec 4. PMID: 27915483.

| **Category of study designs** | **Methodological quality criteria** | **Responses** | | |
| --- | --- | --- | --- | --- |
|  |  | Yes | No | Can’t tell |
| Screening questions (for all types) | S1. Are there clear research questions? | X |  |  |
|  | S2. Do the collected data allow to address the research questions? | X |  |  |
|  | *Further appraisal may not be feasible or appropriate when the answer is ‘No’ or ‘Can’t tell’ to one or both screening questions.* | | | |
| 3. Quantitative non-randomized | 3.1. Are the participants representative of the target population? | X |  |  |
|  | 3.2. Are measurements appropriate regarding both the outcome and intervention (or exposure)? | X |  |  |
|  | 3.3. Are there complete outcome data? | X |  |  |
|  | 3.4. Are the confounders accounted for in the design and analysis? |  |  | X |
|  | 3.5. During the study period, is the intervention administered (or exposure occurred) as intended? | X |  |  |

**Raziano 2017 Comments:**

# The study aimed to determine factors associated with sterilization among HIV-positive women in the United States. The participants were women living with HIV ages 18-45, and were recruited through convenience sampling at an outpatient HIV clinic in the U.S. state of Georgia.

# The measurement of both outcomes and exposures was based on a 225-question survey administered to participants. They were asked about sterilization and factors associated with sterilization such as ART and contraceptive use. The factors (exposures) are relevant to experiences or not of sterilization (outcome).

# Outcome data was complete, and missingness was addressed in the tables. There were two models: Model 1 which excludes variables missing more than 40% of responses, and Model 2 which additionally includes whether a woman had a child born with HIV.

# Confounders were not explicitly addressed in the methodology or results. Adjustment was related to variable inclusion based on missingness, and an additional indicator looking whether a woman had a child born with HIV.

# Data was collected to compare factors associated with sterilization among the participants, and the study type was a retrospective cohort study. The sterilization and other factors (fertility preferences, sexual history) took place before the study period.

# Article Citation: Rice WS, Turan B, Fletcher FE, Nápoles TM, Walcott M, Batchelder A, Kempf MC, Konkle-Parker DJ, Wilson TE, Tien PC, Wingood GM, Neilands TB, Johnson MO, Weiser SD, Turan JM. A Mixed Methods Study of Anticipated and Experienced Stigma in Health Care Settings Among Women Living with HIV in the United States. AIDS Patient Care STDS. 2019 Apr;33(4):184-195. doi: 10.1089/apc.2018.0282. PMID: 30932700; PMCID: PMC6459270.

| **Category of study designs** | **Methodological quality criteria** | **Responses** | | |
| --- | --- | --- | --- | --- |
|  |  | Yes | No | Can’t tell |
| Screening questions (for all types) | S1. Are there clear research questions? | X |  |  |
|  | S2. Do the collected data allow to address the research questions? | X |  |  |
|  | *Further appraisal may not be feasible or appropriate when the answer is ‘No’ or ‘Can’t tell’ to one or both screening questions.* | | | |
| 5. Mixed methods | 5.1. Is there an adequate rationale for using a mixed methods design to address the research question? | X |  |  |
|  | 5.2. Are the different components of the study effectively integrated to answer the research question? | X |  |  |
|  | 5.3. Are the outputs of the integration of qualitative and quantitative components adequately interpreted? | X |  |  |
|  | 5.4. Are divergences and inconsistencies between quantitative and qualitative results adequately addressed? | X |  |  |
|  | 5.5. Do the different components of the study adhere to the quality criteria of each tradition of the methods involved? | X |  |  |

**Rice 2019 Comments:**

1. The research question studies themes around facilitators and barriers to HIV treatment adherence such as stigma, and it also focuses on self-report of HIV stigma and ART adherence. The use of a structured questionnaire (as quantitative methods) is appropriate to assess self-report. Also, the use of in-depth interviews (qualitative methods) is appropriate to provide detailed results or themes around the perceptions and experiences of facilitators and barriers to HIV treatment such as stigma.
2. Both the structured questionnaire and in-depth interviews study facilitators and barriers of HIV stigma differently. The research question initially assessed experiences of women living with HIV through discussion around barriers and facilitators related to HIV treatment adherence (qualitative method). The quantitative method is a follow up from the preliminary findings of the qualitative data; which shows how both methods are integrated to answer the research question.
3. Table 2 shows how quantitative and qualitative are integrated together. Other tables (1,3,4) each show outputs (themes, quotes, odds ratio) from quantitative and qualitative data. For example, quantitative data revealed moderate levels of anticipated stigma for the future (mean = 1.84; range = 1–5) and qualitative quotes about her fear that healthcare workers might not treat her with respect. Interpretation for both components was sound and consistent.
4. The author reported that quantitative findings were consistent with qualitative results. For example, both types of stigma (experienced and anticipated) had differential quantitative effects on ART adherence, based on participants’ age, race, education, income, time on ART, and drug use). There were no inconsistencies noted.
5. Both components (quantitative and qualitative) provided quality inputs and findings for the research question and study. The quotes are relevant to the research question and integrated to the quantitative findings. All components were aligned with quality criteria.

**Article Citation: Strode A, Mthembu S, Essack Z. "She made up a choice for me": 22 HIV-positive women's experiences of involuntary sterilization in two South African provinces. Reprod Health Matters. 2012 Dec;20(39 Suppl):61-9. doi: 10.1016/S0968-8080(12)39643-2. PMID: 23177681.**

| **Category of study designs** | **Methodological quality criteria** | **Responses** | | |
| --- | --- | --- | --- | --- |
|  |  | Yes | No | Can’t tell |
| Screening questions (for all types) | S1. Are there clear research questions? | X |  |  |
|  | S2. Do the collected data allow to address the research questions? | X |  |  |
|  | *Further appraisal may not be feasible or appropriate when the answer is ‘No’ or ‘Can’t tell’ to one or both screening questions.* | | | |
| 1. Qualitative | 1.1. Is the qualitative approach appropriate to answer the research question? | X |  |  |
|  | 1.2. Are the qualitative data collection methods adequate to address the research question? | X |  |  |
|  | 1.3. Are the findings adequately derived from the data? | X |  |  |
|  | 1.4. Is the interpretation of results sufficiently substantiated by data? | X |  |  |
|  | 1.5. Is there coherence between qualitative data sources, collection, analysis and interpretation? | X |  |  |

**Strode 2012 Comments:**

1. The main aim of this research is to obtain in-depth evidence of the experiences of involuntary sterilization of women living with HIV in South Africa. A qualitative approach helped collect more detailed information on the circumstances and experiences of sterilization from participants, and is aligned with precedent for similar inquiries into human rights violations and violence.
2. Data was collected through semi-structured interviews with closed—and open-ended questions. This allowed participants a broader exploration of the interview topic (research question on involuntary sterilization) and an in-depth exploration of data and results.
3. The key issues mentioned in the results were coded from data (transcription from interviews). The results include quotes from participants and the key emerging issues.
4. The discussion highlights challenges (key issues) from women living with HIV in South Africa and other countries. It also reviews issues on consent and sterilization from the participant’s perspective. Interpretation of this qualitative data was logical and could be traced back to the results section.
5. Data was collected from women living with HIV in South Africa who have experienced involuntary sterilization, and the qualitative methodology, thematic analysis, and interpretation show a cohesive and comprehensive inquiry into the holistic experience of involuntary sterilization in this context.

# Article Citation: Towriss CA, Phillips TK, Brittain K, Zerbe A, Abrams EJ, Myer L. The injection or the injection? Restricted contraceptive choices among women living with HIV. Sex Reprod Health Matters. 2019 Dec;27(1):1628593. doi: 10.1080/26410397.2019.1628593. PMID: 31533578; PMCID: PMC6754114.

| **Category of study designs** | **Methodological quality criteria** | **Responses** | | |
| --- | --- | --- | --- | --- |
|  |  | Yes | No | Can’t tell |
| Screening questions (for all types) | S1. Are there clear research questions? | X |  |  |
|  | S2. Do the collected data allow to address the research questions? | X |  |  |
|  | *Further appraisal may not be feasible or appropriate when the answer is ‘No’ or ‘Can’t tell’ to one or both screening questions.* | | | |
| 5. Mixed methods | 5.1. Is there an adequate rationale for using a mixed methods design to address the research question? | X |  |  |
|  | 5.2. Are the different components of the study effectively integrated to answer the research question? | X |  |  |
|  | 5.3. Are the outputs of the integration of qualitative and quantitative components adequately interpreted? | X |  |  |
|  | 5.4. Are divergences and inconsistencies between quantitative and qualitative results adequately addressed? | X |  |  |
|  | 5.5. Do the different components of the study adhere to the quality criteria of each tradition of the methods involved? | X |  |  |

**Towriss 2019 Comments:**

# The research question aims to address the gaps in reproductive intentions and access to contraception for postpartum women living with HIV, by examining the trends and experiences of contraceptive choices and services. The quantitative approach (using longitudinal data) explores the trends and distribution of methods of contraception, and the qualitative approach highlights experiences of women living with HIV in the study. Thus, both methodology approaches are appropriate for the aspect of the research question they help answer.

# The questionnaire and in-depth interviews each explore the research questions differently. The qualitative data collection was among participants from the quantitative data collection, through which participants’ experiences could serve as evidence to what they reported in the questionnaire. The questionnaire supported easy capture of contraception choice and use (the research question).

# The table, figures, and quotes used as outputs are adequate and relevant to the research question and methodology. Also, each type of results is presented clearly: themes for qualitative results and mean, range, and percentages for quantitative results. While discussing the findings, the author highlights how 20% of women were not using any methods of contraception (quantitative results) and how experiences of verbal abuse from HCWs contributed to their contraceptive discontinuation (qualitative results).

# There appear to be no other major inconsistencies between the two study components, with themes from the qualitative inquiry aligning with quantitative data on contraceptive uptake, dynamics, and discontinuation/switching throughout the postpartum period.

# The participants were representative of the target population and were sampled among a cohort of postpartum women with contraception intention and living with HIV or in care. The qualitative analysis highlight themes that are also relevant to the research question for example, familiarity with contraception choices. Hence, both the approaches to the study have provided quality inputs into the study.

**Article Citation: van Dijk, Marieke G. MA, MD; Wilson, Kate S. MPH; Silva, Martha MPH; Contreras, Xipatl BA; Fukuda, Dawn H. ScM; García, Sandra G. ScD. Health Care Experiences of HIV-Infected Women With Fertility Desires in Mexico: A Qualitative Study. Journal of the Association of Nurses in AIDS Care 25(3):p 224-232, May 2014. | DOI: 10.1016/j.jana.2013.04.006**

| **Category of study designs** | **Methodological quality criteria** | **Responses** | | |
| --- | --- | --- | --- | --- |
|  |  | Yes | No | Can’t tell |
| Screening questions (for all types) | S1. Are there clear research questions? | X |  |  |
|  | S2. Do the collected data allow to address the research questions? | X |  |  |
|  | *Further appraisal may not be feasible or appropriate when the answer is ‘No’ or ‘Can’t tell’ to one or both screening questions.* | | | |
| 1. Qualitative | 1.1. Is the qualitative approach appropriate to answer the research question? | X |  |  |
|  | 1.2. Are the qualitative data collection methods adequate to address the research question? | X |  |  |
|  | 1.3. Are the findings adequately derived from the data? | X |  |  |
|  | 1.4. Is the interpretation of results sufficiently substantiated by data? | X |  |  |
|  | 1.5. Is there coherence between qualitative data sources, collection, analysis and interpretation? | X |  |  |

**Van Dijk 2014 Comments:**

1. The qualitative study aimed to explore the experiences of women living with HIV who sought healthcare while pregnant. The qualitative approach using in-depth interviews is appropriate to focus on those experiences.
2. In-depth interviews are necessary to gain insights in the experiences and the dynamics within those situations of the participants. The method was helpful in identifying themes during data collection, analyses, and discussion.
3. The thematic analysis was connected to the data, and findings were also relevant to the research question.
4. The discussion reflects on the themes (results) from the interviews. The discussion also highlights similar results in different countries and similar settings where participants were recruited.
5. The data was collected among participants most likely to have experienced barriers or facilitators in their sexual and reproductive health decision-making, access to healthcare, and quality of care. The discussion and results (themes and quotes) are substantially supported by data, and there was coherence throughout all study components and steps.

# Article Citation: Sukeri S, Sulaiman Z, Hamid NA, Ibrahim SA. Decision-Making on Contraceptive Use among Women Living with Human Immunodeficiency Virus in Malaysia: A Qualitative Inquiry. Korean J Fam Med. 2024 Jan;45(1):27-36. doi: 10.4082/kjfm.23.0088. Epub 2023 Oct 18. PMID: 37848368; PMCID: PMC10822730.

| **Category of study designs** | **Methodological quality criteria** | **Responses** | | |
| --- | --- | --- | --- | --- |
|  |  | Yes | No | Can’t tell |
| Screening questions (for all types) | S1. Are there clear research questions? | X |  |  |
|  | S2. Do the collected data allow to address the research questions? | X |  |  |
|  | *Further appraisal may not be feasible or appropriate when the answer is ‘No’ or ‘Can’t tell’ to one or both screening questions.* | | | |
| 1. Qualitative | 1.1. Is the qualitative approach appropriate to answer the research question? | X |  |  |
|  | 1.2. Are the qualitative data collection methods adequate to address the research question? | X |  |  |
|  | 1.3. Are the findings adequately derived from the data? | X |  |  |
|  | 1.4. Is the interpretation of results sufficiently substantiated by data? | X |  |  |
|  | 1.5. Is there coherence between qualitative data sources, collection, analysis and interpretation? | X |  |  |

**Sukeri 2024 Comments:**

- 1. The study is an inquiry to explore the factors influencing the decisions or decision-making process of women living with HIV to use contraceptive methods. The use of in-depth interviews and focus groups discussion is appropriate, and qualitative methodologies fit the research question.
  2. The in-depth interviews and focus groups help highlight themes or findings for this research question. They provide rich data on a variety of experiences among the study population.
  3. The four themes and selected quotes are relevant to the research question as they are derived from data analysis of the interview transcripts.
  4. Interpretation of results are supported by quotes and themes that are highlighted in the discussion, for example lack of negotiation for condom use and pregnancy at an early age in patriarchal society. Some of the themes are also highlighted in the conclusion.
  5. The interpretations and results (themes and quotes) adequately present the qualitative data (collected through in-depth interviews and focus groups discussions). Each theme highlights different experiences from the participants, and together the study structure is harmonious.

# Article Citation: Sando D, Kendall T, Lyatuu G, Ratcliffe H, McDonald K, Mwanyika-Sando M, Emil F, Chalamilla G, Langer A. Disrespect and abuse during childbirth in Tanzania: are women living with HIV more vulnerable? J Acquir Immune Defic Syndr. 2014 Dec 1;67 Suppl 4(Suppl 4):S228-34. doi: 10.1097/QAI.0000000000000378. PMID: 25436822; PMCID: PMC4251905.

| **Category of study designs** | **Methodological quality criteria** | **Responses** | | |
| --- | --- | --- | --- | --- |
|  |  | Yes | No | Can’t tell |
| Screening questions (for all types) | S1. Are there clear research questions? | X |  |  |
|  | S2. Do the collected data allow to address the research questions? | X |  |  |
|  | *Further appraisal may not be feasible or appropriate when the answer is ‘No’ or ‘Can’t tell’ to one or both screening questions.* | | | |
| 5. Mixed methods | 5.1. Is there an adequate rationale for using a mixed methods design to address the research question? | X |  |  |
|  | 5.2. Are the different components of the study effectively integrated to answer the research question? | X |  |  |
|  | 5.3. Are the outputs of the integration of qualitative and quantitative components adequately interpreted? | X |  |  |
|  | 5.4. Are divergences and inconsistencies between quantitative and qualitative results adequately addressed? | X |  |  |
|  | 5.5. Do the different components of the study adhere to the quality criteria of each tradition of the methods involved? | X |  |  |

**Sando 2014 Comments:**

1. The study analyzes experiences of disrespect and abuse during labor and delivery for women through 1. interviews with postpartum women (n = 2000) 2. direct observation during childbirth (n = 208) 3. structured questionnaires (n = 50), and 4. in-depth interviews (n = 18) with health care providers. Using both qualitative and quantitative methodologies with both healthcare providers and women during childbirth or postpartum, along with direct observation of both types of participants during childbirth, is appropriate for the research question.
2. Both qualitative and quantitative data were collected in parallel and independently. The in-depth interviews of healthcare providers and structured interviews of women who gave birth, provided different insights on the topic of abuse and disrespect during childbirth in Tanzania. Hence, both methods are integrated into each step of answering the research question.
3. Both quantitative and qualitative results are adequately interpreted. The results from structured interviews are highlighted in the tables, whereas results from the in-depth interviews are presented as summaries, quotes, and statistics. Although the results are presented separately, they both highlight different aspects of the research question. Together, the cumulative study components paint a robust picture of the environment, facilitators of disrespect and abuse, and quality of care among women living with HIV and not living with HIV during childbirth at this facility.
4. The authors highlighted the relations between both types of results. For example, none of the women living with HIV reported a breach in confidentiality or abuse due to their HIV status, which may be due to the awareness of healthcare providers in maintaining confidentiality of HIV status and how HIV status did not affect quality of care. Otherwise, there were no inconsistencies and divergences noted.
5. The study samples (all representative of the target populations) were adequate for both aspects of the research question, but were powered differently due to variation in sample size. Nevertheless, the measurements (data collected) through each method were relevant to the research question on disrespect and abuse during childbirth. Also, qualitative results were substantiated and adequately interpreted from the data (for example: providing care for a women living with HIV giving birth and without stigma, and 77% of providers who reported being comfortable providing ART to women during childbirth). All components appeared to adhere to quality criteria.

# Article Citation: Yadzir ZHM, Ramly M, Suleiman A. Do women living with human immunodeficiency virus experience stigma and discrimination when accessing sexual and reproductive health services in government health facilities in Malaysia? J Health Manag. 2023;20(1):96-101

| **Category of study designs** | **Methodological quality criteria** | **Responses** | | |
| --- | --- | --- | --- | --- |
|  |  | Yes | No | Can’t tell |
| Screening questions (for all types) | S1. Are there clear research questions? | X |  |  |
|  | S2. Do the collected data allow to address the research questions? |  | X |  |
|  | *Further appraisal may not be feasible or appropriate when the answer is ‘No’ or ‘Can’t tell’ to one or both screening questions.* | | | |
| 3. Quantitative non-randomized | 3.1. Are the participants representative of the target population? | X |  |  |
|  | 3.2. Are measurements appropriate regarding both the outcome and intervention (or exposure)? |  | X |  |
|  | 3.3. Are there complete outcome data? |  |  | X |
|  | 3.4. Are the confounders accounted for in the design and analysis? |  | X |  |
|  | 3.5. During the study period, is the intervention administered (or exposure occurred) as intended? |  |  | X |

**Yadzir 2023 Comments:**

1. The participants are women living with HIV seeking sexual, reproductive, and postpartum healthcare in government facilities in Malaysia, a group which is representative of the broader target population.
2. A questionnaire (online survey) was used to evaluate stigma and discrimination faced by women living with HIV. However, there is no clear exposure, and the study structure did not comprehensively compare outcomes between two groups. There was disaggregation by current ART use. The types of stigma and discrimination assessed in the survey were appropriate.
3. Table 1 presents results from the data collection. There is no mention of missing data.
4. The data from the study questionnaire was only used for descriptive analysis, not inferential. The analysis did not name confounders or include adjustment.
5. There is no clear exposure or comparison group, as the analysis and study appeared to only use descriptive statistics. Stigma and discrimination rates among the full study population, which occurred within government health facilities before the study period, were the foundation of the study. There was disaggregation of the descriptive findings by current ART use.
