# Supplementary material for: Reproductive coercion experienced by women living with HIV – a global scoping review
Source: Sex Reprod Health Matters. 2026 Feb 3;33(1):2588004. doi: 10.1080/26410397.2025.2588004 (PMC12954806; doi:10.1080/26410397.2025.2588004)
Supplement: Supplementary File A. Search strategy. [file ZRHM_A_2588004_SM7358.docx]

Supplementary File A. Search strategy

Three databases were searched in this review, Embase, PubMed, and LILACS. The last date of search was January 26^th^, 2024. Below is the search terminology and strategy for PubMed.

((women AND (HIV OR AIDS OR HIV+) AND (positive OR living OR diagnosed)) OR WLHIV OR PLHIV) AND ((steriliz* OR tubal ligation) OR "reproductive coerc*" OR “sexual coerc*” OR “medical coerc*” OR "medical abuse" OR abortion OR contracept* OR (cesarean OR c-section) OR ("obstetric violence" OR "maternal violence" OR "obstetric abuse" OR "maternal abuse" OR “labor violence” OR “labor abuse”))

Date slider set to January 1^st^, 2011 or 2011.
