# Supplementary material for: Reproductive coercion experienced by women living with HIV – a global scoping review
Source: Sex Reprod Health Matters. 2026 Feb 3;33(1):2588004. doi: 10.1080/26410397.2025.2588004 (PMC12954806; doi:10.1080/26410397.2025.2588004)
Supplement: Supplementary File C. RC Scoping Review Full-Text Exclusion Criteria. [file ZRHM_A_2588004_SM7356.docx]

Supplementary File C. RC Scoping Review Full-Text Exclusion Criteria

**Section I: Studies that looked promising at title/abstract screening, but turned out to be disqualified**

1. Population does not sufficiently center HIV
2. RC mention was only in abstract or recommendations, but not part of actual study
3. Full text not available (either due to restricted access, translation, or it is just an abstract)

This will most commonly be used for papers that turn out to just be abstracts. If this happens, search for a similarly titled paper, since they may have published a full manuscript after the abstract.

**Section II: Studies that we are interested in because they are very related to RC and HIV, but always planned on excluding at the full text stage**

1. Insufficient focus on reproductive autonomy within clinical setting

Any articles that talk about reproductive coercion or reproductive violence perpetrated by intimate partners would fall under this tag. Articles looking at healthcare worker stigma without mention of coercion or violence could also fall under this tag (or unmet need/fertility desire if those topics are also addressed)

1. Compare contraceptive use, delivery methods, and abortion rates for women not living with HIV and WLHIV without RC or Agency Lens

Comparison of contraceptive usage, discontinuation, and preference, delivery methods (cesarean versus vaginal delivery) and abortion rates MUST look at violence or coercion in order to add value to our final review. These studies look at WLHIV compared to women not living with HIV and will very clearly set up the two populations in the abstract and methods.

1. Fertility Desire, Pregnancy Desire, Family-Planning Desire, Reproductive Decision-Making, or Reproductive Plans for WLHIV without RC or Agency Lens

Fertility desire (and all associated phrases) is a common article topic, and these papers serve as good background for the manuscript introduction and discussion. Studies that were initially included because they looked at healthcare worker perceptions of WLHIV family planning needs may also fall in this tag (as they shouldn’t be included in the final set without mention of coercion). Studies looking at integration of FP and HIV services may also fall here.

1. Method Mix without RC Lens

To catch any method mix papers that slipped through screening by referring to RC.

1. Unmet Need of WLHIV

Unmet need is another common topic and is often included in the title of articles when relevant. Studies that were initially included because they looked at contraceptive choice or access to specific methods may also fall in this tag (as they shouldn’t be included in the final set without mention of coercion). Studies looking at integration of FP and HIV services may also fall here.

**Section III: Studies that are useful for background literature for the review manuscript, but do not qualify for extraction based on format**

1. No novel peer-reviewed findings (systematic reviews, theoretical frameworks, legal reviews, etc)

This should be used for any reproductive coercion literature that are not formal peer-reviewed studies. Most commonly, this will include opinions, viewpoints, and legal reviews.
